# Projections of primary hip arthroplasty in Germany until 2040

**DOI:** 10.1080/17453674.2018.1446463

**Published:** 2018-03-05

**Authors:** Veronika Pilz, Tim Hanstein, Ralf Skripitz

**Affiliations:** 1 Heraeus Medical GmbH, Wehrheim; 2 Universitätsmedizin Rostock, Orthopädische Klinik und Poliklinik, Rostock, Germany; 3 Roland Klinik, Zentrum für Endoprothetik, Fuszchirurgie, Kinder- und Allgemeine Orthopädie, Bremen

## Abstract

**Background and purpose:**

The number of hip replacements in Germany has increased considerably during the last 2 decades but lately levelled off with no significant increase in operation rates. We analyzed the future trend of hip arthroplasty and projected the number of primary hip replacements that will be performed in Germany until 2040.

**Patients and methods:**

We used prevalence data of hip arthroplasty patients from 2010 to 2016 from the nationwide inpatient statistics and population forecasts from the German Federal Bureau of Statistics up to the year 2040. We used Poisson regression to estimate the expected annual number of arthroplasty surgeries with calendar year and patient age as covariates to account for differences among age groups and changes over time.

**Results:**

The number of primary hip replacements performed in Germany in 2040 was estimated to grow by 27% to 288 x 10^3^ (95% CI 250 x 10^3^–332 x 10^3^) from 2010. Projected counts were highest for patients aged 60 to 70 years. The estimated incidence rate was projected to 360 (95% CI 312–414) per 100,000 residents. However, incidence rates for individual age classes were found to be constant with a slight decrease over time for individual age classes.

**Interpretation:**

Our findings suggest a growth in the total hip arthroplasty count whereas incidence rate remained constant over age classes. We consider the future demographic change to an older population as well as the increasing life expectancy to be the main reasons for the increasing patient numbers rather than a general increase in the operation frequency.

Germany is ranked second among the countries of the Organization for Economic Cooperation and Development (OECD) concerning the incidence of hip replacements (OECD 2015). 283 hip arthroplasties per 100,000 residents were performed in 2014 and Germany is thereby at the head of many European countries like Austria, Belgium, and the Nordic countries. The US is a little above average with an incidence of 204 per 100,000 residents. However, incidence rates have increased rapidly in most OECD countries and they are suspected to grow further (OECD 2015). The future rate of hip arthroplasty in Germany might considerably increase with the aging of the baby boomer generation starting to reach 65 in the year 2021 (Nowossadeck 2012). The aging of the population will certainly affect incidences of chronic diseases that are more common in the elderly and in a similar way the inpatient care. At present, hip arthroplasty is 1 of the 10 most commonly performed inpatient procedures in Germany in 2016, which is contributing strongly to the rapidly increasing hospital costs (Statistisches Bundesamt 2017). On the other hand, hospitals face higher constraints on their budgets since DRG reimbursement rates for hip replacement procedures have been reduced. The high frequency of hip replacements in Germany is the subject of ongoing discussion. Therefore, a reliable projection of the demand for future hip replacements would be valuable for decision-makers but also for other stakeholders like insurers or hospitals. There is only a limited amount of data available on the incidence of primary hip replacements in Germany (Schäfer et al. 2013, Wengler et al. 2014) and no study dealing with estimated future demands. Some publications exist outside Germany such as in the US (Kurtz et al. 2007, 2009), the UK (Culliford et al. 2015), and in the Nordic countries (Pedersen et al. 2009, Otten et al. 2010, Nemes et al. 2014, 2015).

In this study, we investigated the epidemiology of hip arthroplasty performed in Germany and projected patient numbers up to the year 2040. We hypothesized that we would observe increasing rates of hip replacements over time and that the rates would vary with respect to the age distribution among the population.

## Methods

### Data

We analyzed data from the nationwide inpatient statistics (DRG statistics), which include treatment data on all inpatient cases processed according to the DRG system. Inpatient episode reporting is mandatory and therefore this study can be considered a nationwide survey (except military and psychiatry services) (Müller-Bergfort and Fritze 2007). In 2016, the DRG statistics included approximately 19 million hospital cases with on average 3.6 procedures (Statistisches Bundesamt 2017). These statistics incorporate anonymized data from the Federal Bureau of Statistics, which undergo plausibility checks and data validations and verifications on a medical and on an economic level (Spindler 2017). The data comprise DRG information including medical procedures but no comprehensive demographic or hospital admission information. Medical procedures are coded according to the national classification of operations and procedures (OPS). It should be mentioned that these hospitalization statistics count admissions, rather than patients. We studied data from the year 2010 up to 2016 and corresponding OPS versions have been used (DIMDI 2016). We identified all patients with a procedure code for hip arthroplasty including either total hip arthroplasty, hemiarthroplasties, or other implant types (Table 1, see Supplementary data). For the years 2010 through 2016, there were no coding changes for hip arthroplasty. In our analysis, all patients were included from birth, which incorporated around 232,000 in 2016. Age was categorized in groups of <45, 45–54, 55–59, 60–64, 65–69, 70–74, 75–79, 80–84, 85–89, and 90 years and above.

Population data were available from the German Federal Bureau of Statistics as well as official statistics of population projection until 2060 (Pötzsch and Röszger 2015) (Figure 1, see Supplementary data). These population projections take into account the future mortality, increased life expectancy for the oldest population groups, and immigration rate. We used the projection model assuming constant trend of birth and death rates with a higher rate of immigration (Figure 2, see Supplementary data). In general, population forecasts are based on hypothetical assumptions about future conditions and factors and are therefore uncertain. Internal validations showed good agreement between predicted and proven results when comparing former German population forecasts since 1998 in the short and medium term with the actual population size and structure (Pötzsch 2016, Statistisches Bundesamt 2016).

### Statistics

We used historical data from 2010 to 2016 and population forecasts up to the year 2040 in order to project the annual incidence of hip replacement surgery in Germany. We used Poisson regression to estimate the expected annual number of arthroplasty surgeries with calendar year and patient age as covariates to account for differences among age groups as well as changes over time. We chose an offset variable for the size of the population to ensure that the operation rate did not rise above the total population number. The regression estimates were limited to grow to the amount of the total population to prevent overestimation. The incidence was calculated by dividing the estimated number of surgical procedures for the national total and for each age subgroup by the corresponding population forecast from the Federal Bureau of Statistics. In order to overcome overdispersion problems that could result in an underestimation of the variance, we used a robust sandwich covariance matrix estimator for variance calculation.

We checked the robustness of our projection and calculated a conservative model for the forecast. As the Poisson regression is assuming continuous growth, the incidence could be modelled to an unreasonably high rate. In the conservative approach, we assumed the THR incidence rate to be constant and thereby only accounted for population changes over time. The incidence of THR procedure is held constant based on the average of historical incidence data from the years 2010 to 2016.

All statistical analyses were performed with R Version 3.4.0 (R Development Core Team, The R Foundation for Statistical Computing, Vienna, Austria).

^Funding and potential conflicts of interest^

The study received no funding. There is no conflict of interest involving any of the authors.

## Results

### Historical view on hip arthroplasty in Germany

Within the DRG statistics data there were approximately 213,000 patients undergoing primary hip arthroplasties in 2010, which increased by 9% to 232,000 patients in the year 2016. Thus, there was a small but steady increase in patient numbers as well as in the incidence rate, which amounted to 283 per 100,000 in 2016 (Table 2). Since 2014, the incidence rate as well as the patient numbers increased by 3% approximately every year.

**Table 2.** Annual number of total population, of hip replacement procedures, and incidence per 100,000 German residents for the years 2010–2016 in Germany

Year	Total population	Cases	Incidence
2010	81,757,471	213,697	261
2011	81,779,210	213,935	262
2012	80,425,879	212,304	264
2013	80,645,665	210,384	261
2014	80,982,525	219,325	271
2015	81,686,633	227,293	278
2016	82,345,000	232,746	283

### Projection of hip arthroplasty in Germany

For modelling the forecasts of the numbers of hip replacements up to the year 2040, the Poisson regression model indicated evidence of overdispersion. The problem of overdispersion could result in underestimating the variance of the estimated parameter. Therefore, we decided to account for overdispersion from the theory of quasi-likelihood and applied a quasi-Poisson regression on our data (Wedderburn 1974).

**Figure 3.**
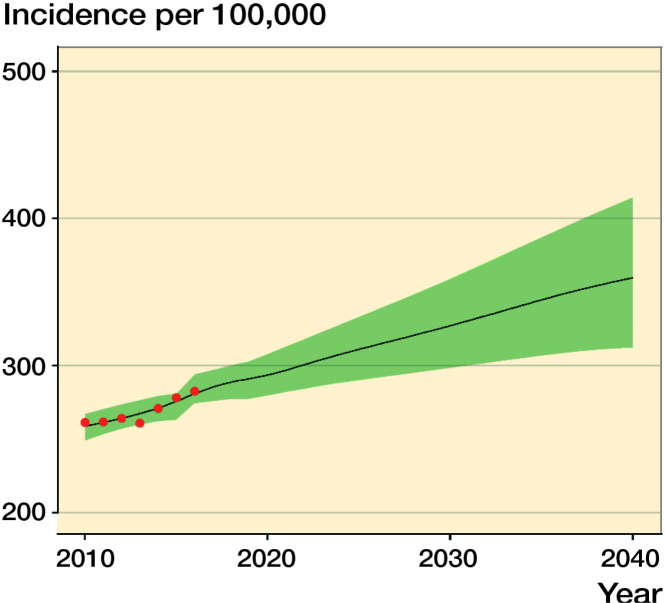
Projected incidence of hip replacements in Germany from 2010 to 2040 with points indicating historical patient numbers. Shading in green indicates the 95% confidence interval.

Under the quasi-Poisson projection model the annual number of hip replacements is forecast to grow to 288 x 10^3^ (95% CI 250 x 10^3^–332 x 10^3^) by 2040. The estimated incidence rate was projected to 360 (95% CI 312–414) per 100,000 German residents, which results in a growth of 38% from 2010 to 2040. Figure 3 shows the projected incidence until 2040 with points representing historical data that were used for setting up the model. Even though variability increases over time, the total incidence rate increases significantly from 2010 to 2040.

**Figure 4.**
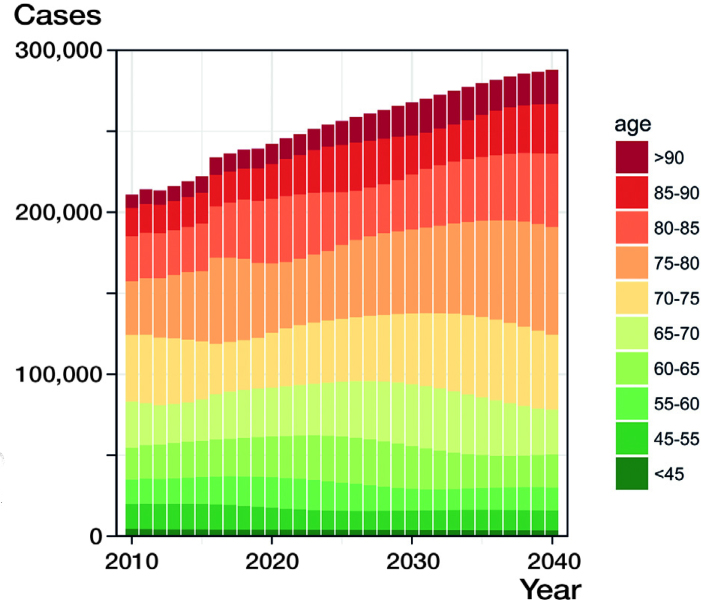
Projected number of hip replacements from 2010 to 2040 by age group.

Figure 4 shows the projections of hip replacement counts for the years 2010 to 2040 colored by age groups. The highest number of patients in 2040 was estimated in the age group 75–80 years, which doubles to up to 66,575 from the year 2010. The highest increase in patients was modelled in the group of patients older than 90, which increased by 160%. On the other hand, hip replacements in patients up to the age of 70 were found to follow a constant or even negative trend. Procedure counts of patients aged 45–55, for instance, decreased by 20% approximately. Overall, it can be concluded that there is a shift towards older patients undergoing hip replacement surgery.

**Figure 5.**
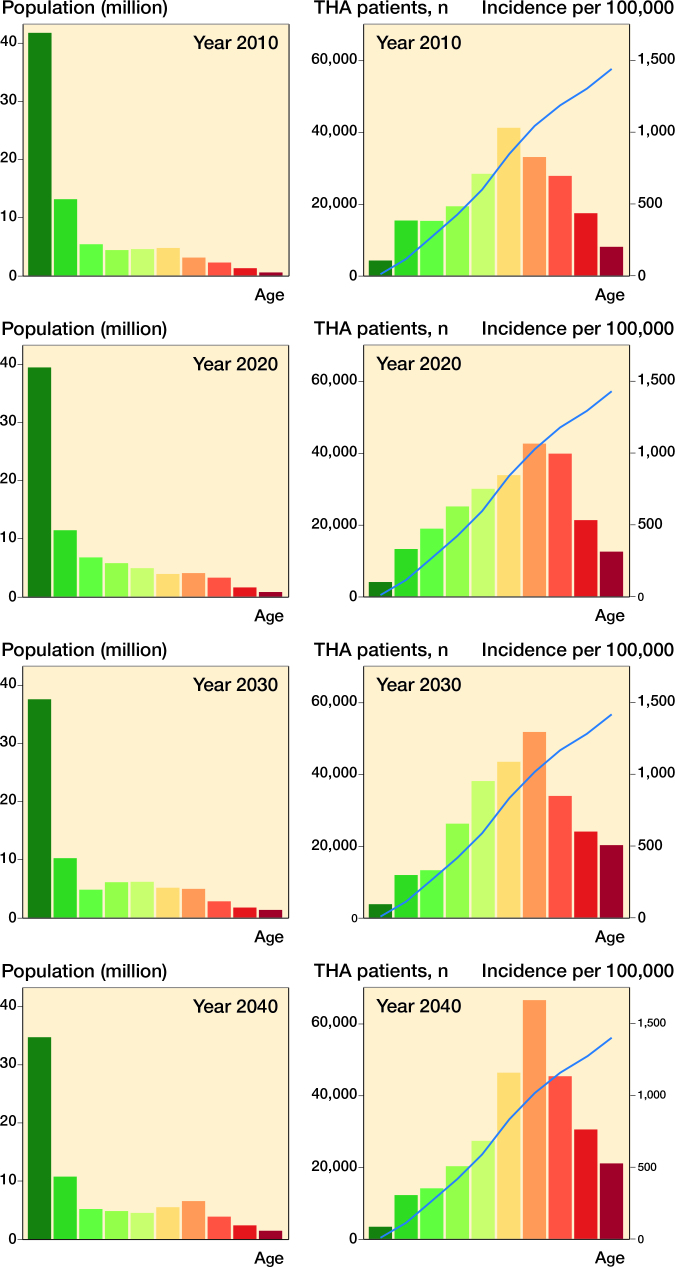
Population forecasts compared with the number of hip replacements and the incidence by age group for the years 2010, 2020, 2030, and 2040. For colour codes, see Figure 4.

In comparison with the increase in the total number of hip replacements or the total incidence, respectively, the incidence per age group is modelled to be constant. In Figure 5 the population forecast is compared with the number of hip replacements and the incidence by age group for the years 2010, 2020, 2030, and 2040. Our model computes the increasing hip replacements count, but in relation to the simultaneously increasing population count the incidence rates per age group are constant over time.

### Sensitivity analysis

Our sensitivity analysis assuming constant incidence rates per age group held fixed by the average of the years 2010–2016 showed similar results to the quasi-Poisson regression. The average total incidence rate was 268 hip replacements per 100,000 German residents. The annual number of THRs was forecast to grow to 295,422 patients in 2040 compared with 287,955 patients calculated by the quasi-Poisson regression (Table 3, see Supplementary data).

## Discussion

### Summary

This epidemiologic study investigated the trend for primary hip arthroplasty in Germany from 2010 to 2040. Given the official population forecasts, the number of primary hip replacements performed in Germany in 2040 was estimated to grow by 27% to 288 x 10^3^ (95% CI 250 x 10^3^ to 332 x 10^3^) from 2010. The estimated incidence rate was projected to rise from 283 in 2016 to 360 (95% CI 312–414) in 2040 per 100,000 German residents. However, incidence rates per age group were modelled to be constant with a slight decrease over time. Furthermore, the increase in hip replacements is mainly based on the trend within different age groups. Whereas a constant or even a negative trend was found for patients aged 70 or younger, patient numbers older than 70 years will increase considerably to 2040.

### Comparison with other German studies

In the literature, only a few publications can be found regarding the trend in hip arthroplasty in Germany. Wengler et al. (2014) examined the current situation over the years 2005–2011 with no projection of hip replacement counts with the use of the DRG statistics data. In their analysis, several inclusion criteria restricted the dataset so that the patient numbers for the years 2010 and 2011 differ slightly from ours. They reported a growth of 11% in elective primary hip replacement operations in Germany from 170 to 190 per 100,000 persons. However, after correction for demographic changes, a 3% increase remained. Thus, Wengler et al. (2014) concluded that the increase in the volume of hip arthroplasty in Germany was most likely attributable to the demographic changes, as we did.

### Comparison with studies from other countries

It has to be mentioned that a direct comparison between countries is complicated by various factors, i.e., different modelling techniques or assumptions and different lifestyle factors as well as social and economic factors influencing hip replacement surgeries. Furthermore, it must be considered that the extent to which crude figures can be interpreted is limited by demographic differences as well as different patient inclusion criteria (elective vs. acute hip fracture surgeries). Therefore, we simply wish to sum up recent findings. In an international comparison the incidence of hip arthroplasty varies considerably, with Germany being among the top countries with the second largest hip replacement incidence behind Switzerland (Pabinger and Geissler 2014, OECD 2015). Similarly, the projected future hip replacements counts vary dependent on the country, whereas all predict the incidence to grow. However, there are few studies describing how future hip arthroplasty incidence might change in future years. In recent years some publications have existed outside Germany, for example in the United States (Kurtz et al. 2007, 2009), the UK (Culliford et al. 2015) or in the Nordic countries (Pedersen et al. 2009, Otten et al. 2010, Nemes et al. 2014, 2015). Kurtz et al. (2007) forecasted the biggest growth of 174% between 2005 and 2030. On the other hand, Culliford et al. (2015) expected an increase of 32% from 2015 to 2035 and a Swedish publication reported only a 25% increase in total hip arthroplasty from 2013 to 2030 (Nemes et al. 2014, 2015). A 66% increase in the incidence rate of primary THAs between 2013 and 2046 is projected for Australia (Inacio et al. 2017). In our study, we modelled an increase of 38% for hip arthroplasties in Germany. Our steady increase in incidence rate appears to be consistent with the reports from other countries. However, the incidence rates per age group were more or less constant over time. Similarly, some publications found a slowing growth rate (Bini et al. 2011, Nemes et al. 2014). For instance, Bini et al. (2011) reported a slowing demand for total joint replacement, with growth rates decreasing from 18% in 2002 to 3% in 2009, and in the Swedish population also the growth of THR decreased after 2000 (Nemes et al. 2014).

### Rising incidences in older age

The incidence of hip osteoarthritis tends to increase with age, which will most likely result in an increasing number of hip arthroplasties. Our study results support this hypothesis as we found the highest number of patients in 2040 in the age group of 75–80 years, which doubled compared with 2010. Pedersen et al. (2009) had similar findings since the incidence rate peaked for patients aged 70–79 during 1996–2002. On the other hand, a study by Pabinger et al. (2014) reported that patients aged 64 or younger have a 7-fold higher growth rate in hip replacement surgeries compared with older patients within the OECD countries. However, a smaller trend was seen for Germany and the categorization of older or younger than 65 might not be suitable for the German population, as it is on average older than other countries. Furthermore, they also identified GDP and health-care expenditures as significant determinants of surgery rates, which could indeed have a greater impact in Germany than in other countries. In other studies a similar trend was found for the United States and in Finland (Kurtz et al. 2009, Skyttä et al. 2011).

### Limitations

Our projection might be limited as it is based on the historical growth of hip arthroplasty counts and the population projection of the Federal Bureau of Statistics. Thus, our projection may be biased from earlier trends inferred from the historical data but these might change in the future. We could take into account the longer life expectancy of the future population by using the official population forecast. On the other hand, we could not adjust for a possible change in lifestyle such as becoming more active in the later phase of life, which would possibly increase the demand for hip replacements. Further factors that might influence the incidence rate are not taken into account such as the trend in available surgeons, new technologies, or techniques making a hip replacement obsolete, economic trends constraining hospital budgets, or new health-care policies. However, there is a lack of data on future need and many of these factors are hard to predict reliably, i.e., when they can only be based on the opinions of a small group of experts. Furthermore, every forecast—even to the near future—bears uncertainties that are hard to predict. Unfortunately, we could only include 7 years of historical primary hip arthroplasty counts due to limited access to the data. It is clear that the regression model would be more robust when relying on a larger database.

When interpreting our results, one major aspect needs to be considered: we could not separate the different kinds of hip replacement surgeries. Thus, our projection includes elective patients as well as patients undergoing acute hip fracture surgery, making our projection population heterogeneous. However, Wengler et al. (2014) examined the share of primary hip replacements for fracture in Germany, which was constant at around 21% during the study period from 2005 to 2011. Furthermore, we checked the OPS codes for hemiarthroplasty, which are predominantly used in acute hip fracture cases, and also saw a constant share over the last years. Consequently, we do not expect a strong rise in hip fractures in the coming years, which would bias our projection model. Nevertheless, when comparing our results with the OECD countries or other publications one has to bear in mind that different patient inclusion criteria may complicate or even hinder a direct comparison.

In our projection model we could only include calendar year and patient age as covariates but did not account for body mass index (BMI), which could also influence the incidence rate of hip arthroplasty (Culliford et al. 2015). However, evidence for BMI as a risk factor for hip osteoarthritis is mixed (Reijman et al. 2007) and it is assumed that the association between BMI and knee osteoarthritis is stronger than for the hip (Jiang et al. 2011).

## Conclusion

Our findings suggest a growth in the total hip arthroplasty count whereas incidence rate remained constant over all age classes. We consider the general future demographic change to an older population as well as the increasing life expectancy to be the main reasons for the increasing patient numbers rather than a general increase in the operation frequency of hip arthroplasty. From an organizational perspective this demographic change might be one of the biggest challenges for orthopedics hospitals over the coming decades to maintain the current standard of care and—to avoid increased waiting times—infrastructure, as well as operation theatre capacity and personnel resources, must follow future demand. For the health-care system the increase in procedures might have a significant financial impact. Furthermore, with the increase in primary replacements, the number of revision procedures is expected to grow as well.  

### Supplementary data

Tables 1 and 3 and Figures 1 and 2 are available as supplementary data in the online version of this article, http://dx.doi.org/10.1080/17453674.2018.1446463

VP: literature review, statistical analysis and interpretation, manuscript draft, revision, and approval. TH, RS: study conception, data collection and data preparation, critical manuscript revision and approval.

*Acta* thanks Ola Rolfson and Eerik T Skyttä for help with peer review of this study.

## Supplementary Material

IORT_A_1446463_SUPP.PDF
