# Supplementary material for: Projections of primary hip arthroplasty in Germany until 2040
Source: Acta Orthop. 2018 Mar 5;89(3):308–13. doi: 10.1080/17453674.2018.1446463 (PMC6055773; doi:10.1080/17453674.2018.1446463)
Supplement: IORT_A_1446463_SUPP.PDF [file IORT_A_1446463_SM0431.pdf]

Supplementary data

Table 1. OPS codes

OPS code	Description
5-820.00	Implantation einer Endoprothese am Hüftgelenk: Totalendoprothese: Nicht zementiert
5-820.01	Implantation einer Endoprothese am Hüftgelenk: Totalendoprothese: Zementiert
5-820.02	Implantation einer Endoprothese am Hüftgelenk: Totalendoprothese: Hybrid (teilzementiert)
5-820.20	Implantation einer Endoprothese am Hüftgelenk: Totalendoprothese, Sonderprothese: Nicht zementiert
5-820.21	Implantation einer Endoprothese am Hüftgelenk: Totalendoprothese, Sonderprothese: Zementiert
5-820.22	Implantation einer Endoprothese am Hüftgelenk: Totalendoprothese, Sonderprothese: Hybrid (teilzementiert)
5-820.50	Implantation einer Endoprothese am Hüftgelenk: Gelenkpfannenstützschale: Nicht zementiert
5-820.51	Implantation einer Endoprothese am Hüftgelenk: Gelenkpfannenstützschale: Zementiert
5-820.70	Implantation einer Endoprothese am Hüftgelenk: Gelenkschnapp-Pfanne: Nicht zementiert
5-820.71	Implantation einer Endoprothese am Hüftgelenk: Gelenkschnapp-Pfanne: Zementiert
5-820.72	Implantation einer Endoprothese am Hüftgelenk: Gelenkschnapp-Pfanne: Hybrid (teilzementiert)
5-820.80	Implantation einer Endoprothese am Hüftgelenk: Oberflächenersatzprothese: Nicht zementiert
5-820.81	Implantation einer Endoprothese am Hüftgelenk: Oberflächenersatzprothese: Zementiert
5-820.82	Implantation einer Endoprothese am Hüftgelenk: Oberflächenersatzprothese: Hybrid (teilzementiert)
5-820.x0	Implantation einer Endoprothese am Hüftgelenk: Sonstige: Nicht zementiert
5-820.x1	Implantation einer Endoprothese am Hüftgelenk: Sonstige: Zementiert
5-820.x2	Implantation einer Endoprothese am Hüftgelenk: Sonstige: Hybrid (teilzementiert)
5-820.y	Implantation einer Endoprothese am Hüftgelenk: N.n.bez.
5-820.30	Implantation einer Endoprothese am Hüftgelenk: Femurkopfprothese: Nicht zementiert
5-820.31	Implantation einer Endoprothese am Hüftgelenk: Femurkopfprothese: Zementiert
5-820.40	Implantation einer Endoprothese am Hüftgelenk: Duokopfprothese: Nicht zementiert
5-820.41	Implantation einer Endoprothese am Hüftgelenk: Duokopfprothese: Zementiert
5-820.92	Implantation einer Endoprothese am Hüftgelenk: Kurzschaft-Femurkopfprothese: Ohne Pfannenprothese, nicht zementiert
5-820.93	Implantation einer Endoprothese am Hüftgelenk: Kurzschaft-Femurkopfprothese: Ohne Pfannenprothese, zementiert
5-820.94	Implantation einer Endoprothese am Hüftgelenk: Kurzschaft-Femurkopfprothese: Mit Pfannenprothese, nicht zementiert
5-820.95	Implantation einer Endoprothese am Hüftgelenk: Kurzschaft-Femurkopfprothese: Mit Pfannenprothese, zementiert
5-820.96	Implantation einer Endoprothese am Hüftgelenk: Kurzschaft-Femurkopfprothese: Mit Pfannenprothese, hybrid (teilzementiert)

Table 3. Annual number of the total population, of the hip replacement procedures and its incidence per 100,000 German residents for the years 2010, 2016, 2020, 2030 and 2040 in Germany for the main and the conservative model

		Main model		Conservative model	
Year	Total population	Cases	Incidence	Cases	Incidence
2010	81,757,471	210,848	258	210,681	258
2016	82,345,000	233,862	284	234,977	285
2020	82,594,000	242,291	293	244,177	296
2030	81,861,000	267,788	327	272,262	333
2040	80,061,000	287,955	360	295,422	369

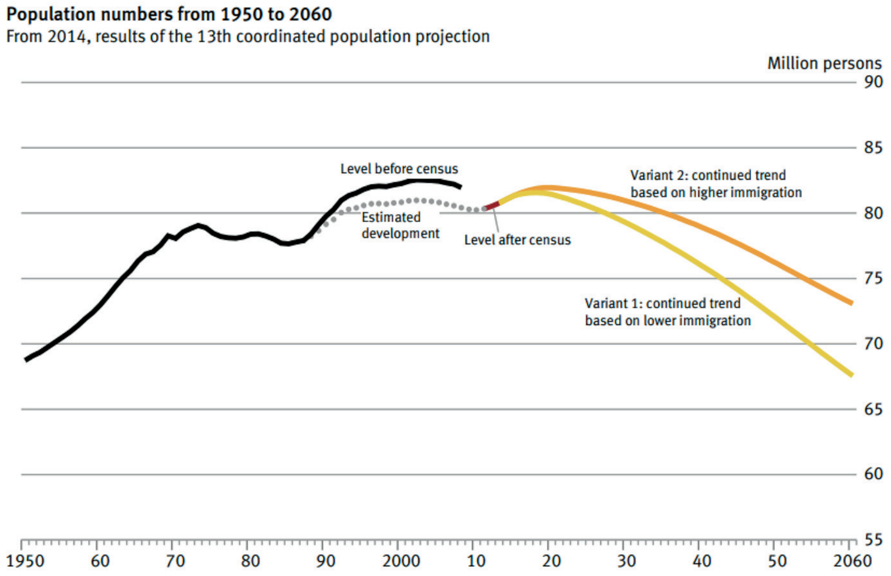

Figure 1. Population numbers from 1950 to 2060, results of the 13th coordinated population projection. Variant 2: continued trend based on higher immigration (Pötzsch and Rößger 2015).

Table 2: Development of Germany's population by 2060 1)
Variant 2: continued trend based on higher immigration

Birth rate of 1.4 children per woman; life expectancy at birth in 2060: 84.8 years for boys/88.8 years for girls; long-term net migration: 200,000 (G1-L1-W2)

Specification		31 Dec. of the year					
		2013	2020	2030	2040	2050	2060
		**Age limits 20 and 60 years**					
**Population, total**	1000....	80 767	81 953	80 919	78 906	76 115	73 079
	2013 = 100....	100	101,5	100,2	97,7	94,2	90,5
under 20 years	1000....	14 684	14 411	14 240	13 271	12 308	11 989
	%....	18,2	17,6	17,6	16,8	16,2	16,4
	2013 = 100....	100	98,1	97,0	90,4	83,8	81,6
20 to under 60 years	1000....	44 137	43 349	38 655	37 470	35 186	33 163
	%....	54,6	52,9	47,8	47,5	46,2	45,4
	2013 = 100....	100	98,2	87,6	84,9	79,7	75,1
60 years and older	1000....	21 946	24 192	28 024	28 165	28 621	27 926
	%....	27,2	29,5	34,6	35,7	37,6	38,2
	2013 = 100....	100	110,2	127,7	128,3	130,4	127,3
**Young-age, old-age, total dependency ratios**							
Per one hundred 20 to under 60 year olds there are							
under 20 year olds..........		33,3	33,2	36,8	35,4	35,0	36,2
60 year olds and older..		49,7	55,8	72,5	75,2	81,3	84,2
together		83,0	89,1	109,3	110,6	116,3	120,4
		**Age limits 20 and 65 years**					
**Population, total**	1000....	80 767	81 953	80 919	78 906	76 115	73 079
	2013 = 100....	100	101,5	100,2	97,7	94,2	90,5
under 20 years	1000....	14 684	14 411	14 240	13 271	12 308	11 989
	%....	18,2	17,6	17,6	16,8	16,2	16,4
	2013 = 100....	100	98,1	97,0	90,4	83,8	81,6
20 to under 65 years	1000....	49 232	49 194	44 831	42 280	40 642	37 909
	%....	61,0	60,0	55,4	53,6	53,4	51,9
	2013 = 100....	100	99,9	91,1	85,9	82,6	77,0
65 years and older	1000....	16 851	18 348	21 848	23 355	23 166	23 181
	%....	20,9	22,4	27,0	29,6	30,4	31,7
	2013 = 100....	100	108,9	129,7	138,6	137,5	137,6
**Young-age, old-age, total dependency ratios**							
Per one hundred 20 to under 65 year olds there are							
under 20 year olds..........		29,8	29,3	31,8	31,4	30,3	31,6
65 year olds and older..		34,2	37,3	48,7	55,2	57,0	61,1
together		64,1	66,6	80,5	86,6	87,3	92,8
		**Age limits 20 and 67 years**					
**Population, total**	1000....	80 767	81 953	80 919	78 906	76 115	73 079
	2013 = 100....	100	101,5	100,2	97,7	94,2	90,5
under 20 years	1000....	14 684	14 411	14 240	13 271	12 308	11 989
	%....	18,2	17,6	17,6	16,8	16,2	16,4
	2013 = 100....	100	98,1	97,0	90,4	83,8	81,6
20 to under 67 years	1000....	50 957	51 251	47 437	44 036	42 636	39 789
	%....	63,1	62,5	58,6	55,8	56,0	54,4
	2013 = 100....	100	100,6	93,1	86,4	83,7	78,1
67 years and older	1000....	15 126	16 291	19 242	21 598	21 171	21 301
	%....	18,7	19,9	23,8	27,4	27,8	29,1
	2013 = 100....	100	107,7	127,2	142,8	140,0	140,8
**Young-age, old-age, total dependency ratios**							
Per one hundred 20 to under 67 year olds there are							
under 20 year olds..........		28,8	28,1	30,0	30,1	28,9	30,1
67 year olds and older..		29,7	31,8	40,6	49,0	49,7	53,5
together		58,5	59,9	70,6	79,2	78,5	83,7

1) From 2020 estimates of the 13th coordinated population projection.
Discrepancies in totals are due to rounding.

Figure 2. Development of Germany's population to 2060 by variant 2: continued trend based on higher immigration (Pötzsch and Rößger 2015).
